# *Tox*-positive *Corynebacterium ulcerans* in hedgehogs, Germany^*^

**DOI:** 10.1080/22221751.2018.1562312

**Published:** 2019-01-21

**Authors:** Anja Berger, Alexandra Dangel, Martin Peters, Kristin Mühldorfer, Silke Braune, Tobias Eisenberg, Claudia A. Szentiks, Jörg Rau, Regina Konrad, Stefan Hörmansdorfer, Nikolaus Ackermann, Andreas Sing

**Affiliations:** a National Consiliary Laboratory for Diphtheria, Oberschleißheim, Germany; b MA DTM&H, National Consiliary Laboratory for Diphtheria, Bavarian Health and Food Safety Authority, Oberschleißheim, Germany; c Chemisches und Veterinäruntersuchungsamt Westfalen, Arnsberg, Germany; d Leibniz Institute for Zoo and Wildlife Research, Berlin, Germany; e Lower Saxony State Office for Consumer Protection and Food Safety, Food and Veterinary Institute, Hannover, Germany; f Hessian State Laboratory (LHL), Gießen, Germany; g Chemisches und Veterinäruntersuchungsamt Stuttgart, Fellbach, Germany

**Keywords:** Tox-positive, *Corynebacterium*, ulcerans, hedgehogs, Germany

## Abstract

Toxigenic *Corynebacterium ulcerans* may cause both respiratory and cutaneous diphtheria in humans. As a zoonotic emerging pathogen it has been isolated from a wide variety of animals living in captivity, such as livestock, pet, zoo and research animals and additionally in a large number of different wild animals. Here we report the isolation of *tox*-positive *C. ulcerans* in four hedgehogs with cutaneous diphtheria and pneumonia, respectively.

## Introduction

Diphtheria and diphtheria-like illness are caused by *Corynebacterium* species harbouring the diphtheria toxin (DT) encoding *tox* gene. In recent years, diphtheria-like human infections with toxigenic *Corynebacterium ulcerans* have outnumbered those caused by toxigenic *C. diphtheriae* in many industrialized countries [1–3]. While about 50 years ago human cases of *C. ulcerans*-caused disease were associated with consumption of raw milk and dairy products or contact to cattle [3–5], nearly all *C. ulcerans* infections since then have been described after contact with domestic animals such as pet dogs and cats [3,6–11] or – less often – after occupational contact with livestock animals such as pigs [12,13]. Moreover, both non-toxigenic and toxigenic *C. ulcerans* as emerging zoonotic pathogens have been isolated from a wide variety of animal species, either from zoo, shelter, research or herd animals with human contact, e.g. water rats [14], shelter dogs [15,16], macaques [17,18], killer whales [19], a lion [19], a dromedary [20], ferrets [21], a goat [22], a cow [23] and ground squirrels [24] or from free-roaming animals such as otters [25], roe deer [26,27], wild boars [27,28], red fox [29], Ural owl [30] and Japanese shrew-moles [30] (Table 1). Interestingly, most of the toxigenic *C. ulcerans* strains were found either in carnivores or animals with (seasonal) group hierarchical fighting. Here we report on the unusual finding of toxigenic *C. ulcerans* in four hedgehogs (*Erinaceus europaeus*), three of them without known previous contact to humans.

**Table 1.** Characteristics of *Corynebacterium ulcerans* isolated from free-roaming wild animals.

Animal species	Number	Country	Clinical manifestation	Source	Toxigenicity	Reference
Otter (*Lutra lutra*)	2	Great Britain (Scotland and England)	Found dead; lung damage	Lung biopsies	Toxigenic	[25]
Roe deer (*Capreolus capreolus*)	1	Southern Germany	Abscess	Abscess material	NTTB	[26,27]
Wild boar (*Sus scrofa*)	12	Southern, Western and North-Eastern Germany	Abscesses and enlarged lymph nodes	Abscess and/or lymph node material	NTTB	[27,28]
Red fox (*Vulpes vulpes*)	1	Southern Germany	Distemper	Splenic tissue	Toxigenic	[29]
Ural owl (*Strix uralensis*)	1	Japan	Asymptomatic	Throat swab	Toxigenic	[30]
Japanese shrew-mole *(Urotrichus talpoides*)	2	Japan	Asymptomatic	Throat swab	Toxigenic	[30]
Hedgehog (*E. europaeus)*	1	Western Germany	Deep soft tissue wound	Wound swab	Toxigenic	Current paper
Hedgehog (*E. europaeus)*	3	Eastern and Northern Germany	Pneumonia, bacteriemia	Lung tissue, heart tissue	1 NTTB 2 toxigenic	Current paper

NTTB non-toxigenic *tox*-bearing.

## Results

**Figure 1.**
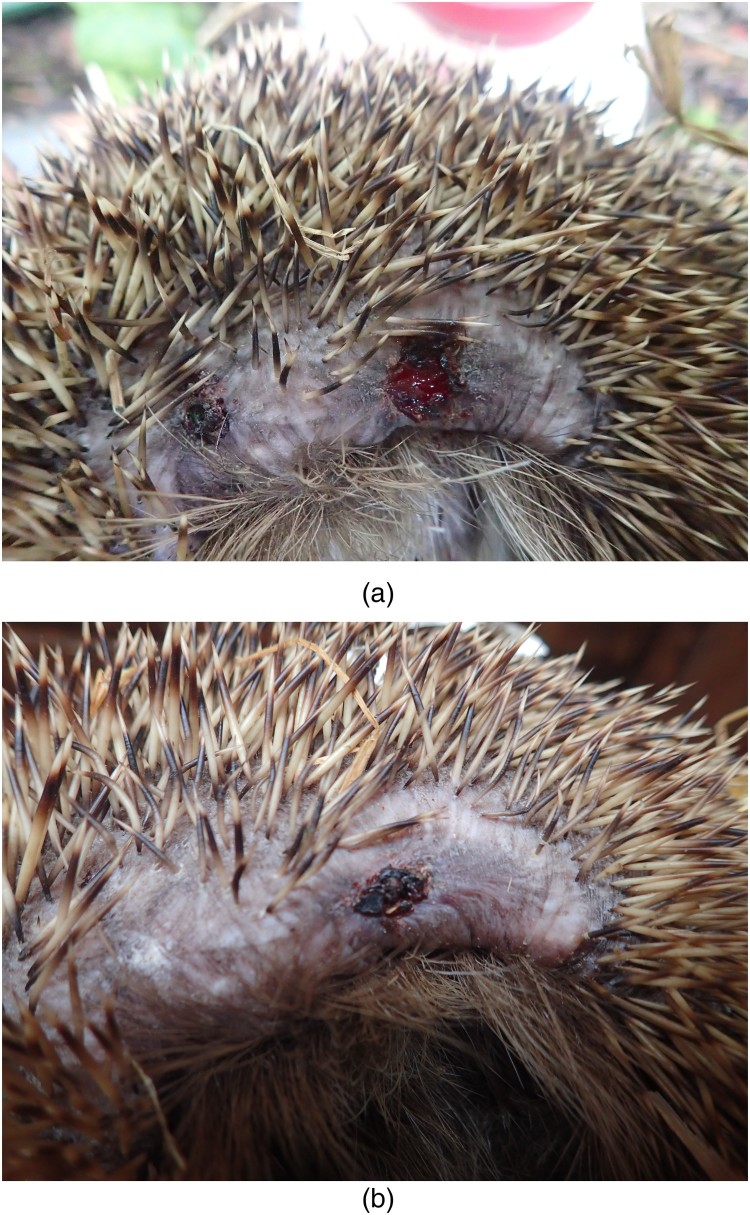
(a,b) Wound infection due to toxigenic *C. ulcerans* in a hedgehog, healing progress under antibiotic treatment.

In December 2017, a young hedgehog (#1) was found with a weight of 1026 g in a garden with severe soft tissue damage after being cut by a mowing machine (Figure 1(a)). The injured animal was brought to a local veterinarian and treated for 12 days with enrofloxacin and a proteinolytic ointment. Because of an extremely retarded wound healing and severe loss of weight the animal was transferred to a private hedgehog rescue station in March 2018, where the animal (750 g) was presented to another veterinarian and taken care of. A wound swab was taken for bacteriological diagnosis. The animal was treated with a third generation cephalosporin which was later switched to sulphonamides for 10 days. The wound continued to heal within three months (Figure 1(b,c)) with diminishing necrotic wound margins.

In April 2017, a male hedgehog (#2) was found in an allotment colony in Berlin, Germany, in a moribund condition. The animal died and was subjected to necropsy showing a weight of 570 g but considering the adipose tissue still had a good nutritional status. Gross pathological examination revealed otitis externa (left ear), anaemia and severe tick infestation. Thickening and redness of the lung suggestive of pneumonia were observed prompting further histological and bacteriological investigations.

In July 2018 two hedgehogs (#3 and #4) were found in Hanover, Germany and finally euthanized because of moribund conditions. Both were adult, male animals with a weight of 760 and 605 g, respectively. Pathological examination revealed myiasis and otitis externa in both cases. Further histological and bacteriological investigations were done because of gross pathological aspects of severe pneumonia and septicaemia in both cases. None of the four hedgehogs presented typical local or systemical findings of diphtheria toxin effects such as pseudomembranes or histopathological lesions indicating myocarditis or damage of the peripheral nervous system.

The wound swab obtained from the severe soft tissue wound of hedgehog #1 grew *C. ulcerans* (strain number KL 1151) and *Streptococcus pyogenes.* Toxigenicity was verified by real-time PCR and a modified Elek test both yielding positive results. Histopathological examination of lung tissues of hedgehogs #2, #3 and #4 showed pneumonia and a severe lungworm infection in hedgehog #2, respectively. Lung tissue material obtained from hedgehog #2 grew *C. ulcerans* (strain number KL 955) in pure culture. Toxigenicity testing by *tox*-PCR and Elek identified the isolate as non-toxigenic *tox*-bearing (NTTB). Lung and heart tissue material obtained from hedgehog #3 grew *Enterococcus avium, Morganella morganii and C. ulcerans* (KL 1203). *C. ulcerans* strain KL 1204 was isolated in pure culture from heart and lung tissue materials obtained from hedgehog #4. Both KL 1203 and KL 1204 were toxigenic as shown by positive *tox*-PCR and Elek testing, respectively. All *C. ulcerans* were identified by partial *rpoB* sequencing, FT-IR and MALDI-TOF analysis.

Commercially available biochemistry systems unequivocally identified all four isolates as *C. ulcerans* (VITEK, Omnilog) with the exception of isolate KL 955 which was falsely identified as *C. pseudotuberculosis* by VITEK CBC. All isolates were found to be resistant against penicillin (MICs 0.19–0.25 mg/l) and clindamycin (MICs 2–4 mg/l) according to EUCAST, but susceptible against erythromycin, cephalosporins and sulphonamides according to CLSI guidelines. NGS-derived MLST based on seven housekeeping loci was performed using NGS data and revealed three different sequence types (ST), 332 in hedgehog #1 (KL 1151) and hedgehog #4 (KL 1204), ST 330 in hedgehog #2 (KL 955), and ST 331 in hedgehog #3 (KL 1203), respectively.

**Figure 2.**
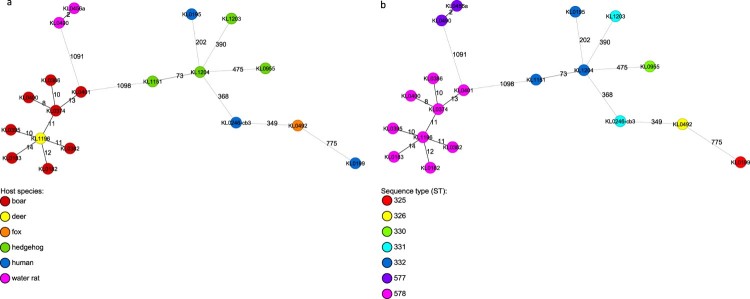
(a,b) Phylogenetic minimum spanning trees of the cgMLST analysis of 19 *C. ulcerans* isolates originating from various host species with an in-house *C. ulcerans*-specific cgMLST scheme of 1211 target loci. Allele distances between samples are indicated. Samples are colour coded by the corresponding host organism (A) or by their ST based on the 7-gene scheme (B), as given in the legend.

Phylogenetic minimum spanning trees, built from cgMLST results of NGS data showed that the genetic similarity of the four *C. ulcerans* isolates from hedgehogs was much lower to the NTTB wildlife cluster from wild boars and roe deer (>1000 alleles) than to human samples from different geographic regions (>200 alleles). However, genomic differences in cgMLST analysis were at least 73 alleles between isolates from hedgehog #1 and #4 which shared the same ST 332 based on the 7-gene scheme and more than 200 alleles compared to all other isolates. These differences show that the hedgehog-derived isolates are genetically not closely related to each other or to any other human or animal isolate (Figure 2(a,b)).

**Figure 3.**
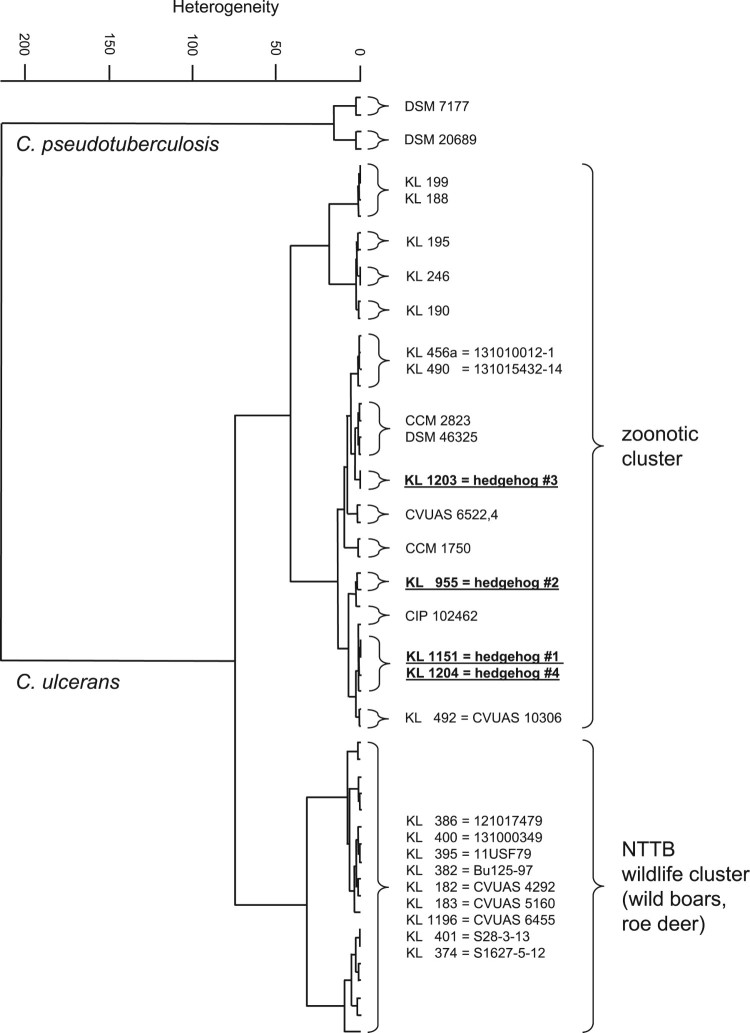
Dendrogram of FT-IR-spectra of *C. ulcerans* strains obtained from the four hedgehogs (underlined) in comparison with spectra from *C. ulcerans* DSM 46325 and several *C. ulcerans* isolates, including isolates from wild animals and humans in Germany. Spectra of two *C. pseudotuberculosis* strains are used as outgroup.

The comparison of FT-IR spectra (Figure 3) shows no similarity for the four hedgehog isolates with the NTTB wildlife cluster (wild boars, roe deer) observed in different parts of Germany [26–28].

## Discussion

In contrast to the classical diphtheria agent *C. diphtheriae* which is basically a human pathogen and has only extremely rarely been reported to be isolated from animals [32], the emerging pathogen *C. ulcerans* is a zoonotic pathogen with an increasing spectrum of affected animals. While originally only reported from livestock (cattle, pigs) and pet (dog, cat) animals, *C. ulcerans* has been meanwhile detected in a wide variety of species living in captivity as zoo (killer whales, lion, water rats), shelter (dogs), herd (dromedary, goat, cow) or research (macaques, ground squirrels) animals with contact to humans. In recent years, isolation of *C. ulcerans* has also been reported in wildlife (Table 1). Interestingly, the broad majority of wild animals affected by *C. ulcerans* showed pathologic lesions of internal organs such as lymph nodes [26–28], lung [25] – also in hedgehogs #2, #3 and #4 of the current study – or spleen [29] suggesting systemic infection. These findings are in contrast to human diphtheria cases due to *C. diphtheriae* or *C. ulcerans* exhibiting respiratory or cutaneous manifestations or to *C. ulcerans* infections in animals living in captivity which were reported to be either asymptomatic carriers or to present with skin or mucosal ulceration. One could assume that only wild animals with a deteriorating disease, possibly aggravated by *C. ulcerans* infection are found and diagnosed, while the asymptomatic carriership of *C. ulcerans* in wildlife is likely as usual as in other animals. Asymptomatic pharyngeal carriage is known for *C. diphtheriae* and – rarely – *C. ulcerans* in humans, but also for *C. ulcerans* primarily in pets and less frequently in livestock animals. In wildlife, however, it has so far only been reported in a recent surveillance study among wild birds and their prey animals [30]. Most reported *C. ulcerans* strains in animals – both with and without human contact – harbour the *tox* gene [10]. Further studies are needed to determine if this is only a reporting bias or reflects the real distribution of toxigenic and non-toxigenic *C. ulcerans* strains among animals and also humans. Since DT producing strains among wild animals were until very recently detected only in carnivores (otters, red fox, Ural owl) with non-toxigenic strains isolated from omnivorous (e.g. wild boars) and herbivorous (e.g. roe deer) animals, it seemed possible that *C. ulcerans* toxigenicity might be associated with a carnivorous lifestyle involving predatory hunting behaviour with the potential of acquiring an infection while fighting. However, the recent detection of asymptomatic carriage of toxigenic *C. ulcerans* in two Japanese shrew-moles [30], as well as the current cases of hedgehogs (this study), broadens the spectrum of affected animals also to primarily insectivorous species.

In contrast to the majority of reported *C. ulcerans* infections in humans causing cutaneous diphtheria [33] as well as in pet or livestock animals [3] with mucosal or skin involvement, superficial soft tissue infection in wild animals has so far only been reported in the current hedgehog #1. Similar to most cases of human cutaneous *C. diphtheriae*-caused diphtheria the infection in hedgehog #1 was associated with a previous trauma, but the source of *C. ulcerans* remains unclear. The strain might be acquired from the environment, an anatomical site of the hedgehog, during its stay in the hedgehog rescue station or from another carnivorous animal trying to feed on the heavily injured hedgehog. However, no signs of animal-afflicted bite wounds were noticed. In hedgehog #3 and #4 otitis externa induced by myiasis could possibly be the portal of entry for the *C. ulcerans* strains.

Notably, according to the recently revised German recommendations [31], public health measures including personal protection, antibiotic prophylaxis and screening for *C. ulcerans* carriage for close contact persons were advised, since zoonotic transmission from pet animals to humans has been clearly demonstrated using molecular typing techniques [6,8–12]. The analysed dataset also indicates closer genetic similarity of the hedgehog-derived isolates to human isolates than to those from wild animals, although no close relationship of the hedgehog isolates to any other isolate was detected. However, *C. ulcerans* carrier status of persons with direct contact to hedgehogs was not a subject of the investigation. As a bacteriological examination with detection of *C. ulcerans* was performed after intensive care treatment of hedgehog #1, all close contact persons refused recommended measures and only engaged in hygienic behaviour and self-observation for clinical signs of diphtheria, raising the general awareness of zoonotic agents in wildlife care. Toxigenic *C. ulcerans* harbour either prophages or, an alternative pathogenicity island (PAI) described previously and can therefore act as a beta corynephage reservoir [10].

In conclusion, the finding of toxigenic *C. ulcerans* in hedgehogs, an increasingly synanthropic species known to reside in urban and suburban environments in close proximity to humans, highlights potential transmission risks and should raise the public health awareness towards zoonotic infections.

## Material and methods

For bacteriological examination, clinical material obtained from all four animals (i.e. wound swabs and lung or heart tissue, respectively) were plated on Columbia agar with 5% sheep blood, chocolate agar supplemented with Vitox (5% CO_2_ atmosphere) and Gassner agar (Oxoid, Wesel, Germany) and incubated for up to 48 hours at 37 °C. Bacteriological species identification was performed as recently described [34] using MALDI-TOF MS analysis (Microflex LT Mass Spectrometer, MALDI Biotyper™; Bruker Daltonics, Bremen, Germany) and the MBT 7311 commercial library. Supplementary species identification by commercial biochemistry assays (VITEK2-compact with card systems for anaerobes and corynebacteria [ANC] and coryneform bacteria [CBC; all bioMérieux, Nürtingen, Germany] and Omnilog [Biolog, Hayward, USA]) was done according to the manufactureŕs prescriptions. Fourier-transform infrared (FT-IR) spectroscopy with cluster analysis, and partial sequencing of the *rpoB* gene were carried out as described previously [27,28,35]. Toxigenicity was investigated by real-time PCR [36] and a modified Elek test [37]. Next-generation sequencing (NGS) of the isolates was performed on an Illumina MiSeq (Illumina, San Diego, CA, USA) as reported previously [38]. Multilocus sequence typing (MLST) based on seven housekeeping loci [39] was done using the NGS data. The sequence type (ST) was determined with the respective MLST database (http://pubmlst.org/cdiphtheriae/). For cg (core genome) MLST typing an ad-hoc *C. ulcerans*-specific cgMLST scheme was generated by using the SeqSphere+ target definer tool (Ridom, Munster, Germany) with default settings [40]. As a reference, the genome of strain 809 with accession number NC_018101 was used. 11 complete *C. ulcerans* genomes from NCBI were used as query sequences for core genome scheme definition (accession nos. NC_018101.1, NZ_CP009716.1, NZ_CP010818.1, NZ_CP011095.1, NZ_CP009583.1, NZ_CP009500.1, NC_015683.1, NZ_CP009622.1, NZ_CP011913.1, NZ_LT906443.1, NZ_CP021417.1). The resulting cgMLST scheme consisted of 1,211 target loci. cgMLST with the described ad-hoc scheme was performed using NGS data as described [38]. NGS raw datasets are available in the NCBI sequence read archive (SRA) at https://www.ncbi.nlm.nih.gov/sra (accession numbers in Supplementary Table 1). Antibiotic susceptibility testing was performed according to both CLSI (CLSI: Performance standards for antimicrobial susceptibility testing. M100, 28th. Ed., Jan 2018;

CLSI: Methods for Antimicrobial Dilution and Disk Susceptibility Testing of infrequently isolated or fastidious bacteria. M45 3rd Ed, 2015) and EUCAST guidelines (http://www.eucast.org/clinical_breakpoints, version 8.1).

For histopathological examination, small slices of lung tissue were fixed in 4% buffered formalin, processed using standard methods and embedded in liquid paraffin. Sections were stained with hematoxylin-eosin (HE).

## Summary of the conclusions

The first isolation of *tox*-positive *Corynebacterium ulcerans* from four hedgehogs underlines both the veterinary and the human public health importance of a variety of wild animals which might serve as zoonotic *C. ulcerans* reservoirs for pet or livestock animals and humans.
